# Duodenal Trauma: Mechanisms of Injury, Diagnosis, and Management

**DOI:** 10.3390/jcm15020567

**Published:** 2026-01-10

**Authors:** Raffaele Bova, Giulia Griggio, Serena Scilletta, Federica Leone, Carlo Vallicelli, Vanni Agnoletti, Fausto Catena

**Affiliations:** 1 General, Emergency and Trauma Surgery Department, Bufalini Hospital, 47521 Cesena, Italy; raffaele.bova@auslromagna.it (R.B.); giulia.griggio@outlook.it (G.G.); serenascilletta@gmail.com (S.S.); fede.leone1990@gmail.com (F.L.); fausto.catena@auslromagna.it (F.C.); 2 Anesthesia, Intensive Care and Trauma Department, Bufalini Hospital, 47521 Cesena, Italy; vanni.agnoletti@auslromagna.it; 3 Department of Medical and Surgical Sciences, Bologna University, 40138 Bologna, Italy

**Keywords:** duodenal injuries, trauma, perforation, non-operative management, primary repair, damage control surgery

## Abstract

**Background**: Traumatic injuries of the duodenum are generally rare but when they occur, they can result in serious complications. Inaccurate injury classification, delayed diagnosis, or late treatment can significantly raise morbidity and mortality. A multidisciplinary approach is often necessary. **Mechanisms of injury**: Isolated duodenal injuries are relatively uncommon due to the duodenum’s proximity to pancreas and major vascular structures. Duodenal injuries can result from blunt or penetrating trauma. **Classification**: The 2019 World Society of Emergency Surgery (WSES)-American Association for the Surgery of Trauma (AAST) guidelines recommend incorporating both the AAST-OIS grading and the patient’s hemodynamic status to stratify duodenal injuries into four categories: Minor injuries WSES class I, Moderate injuries WSES class II, Severe injuries WSES class III, and WSES class IV. **Diagnosis**: The diagnostic approach involves a combination of clinical assessment, laboratory investigations, radiological imaging and, in particular situations, surgery. Prompt diagnosis is critical because delays exceeding 24 h are associated with a higher incidence of postoperative complications and a significant rise in mortality. Contrast-enhanced abdominal computed tomography (CT) represents the gold standard for diagnosis in patients who are hemodynamically stable. **Management**: Duodenal trauma requires a multimodal approach that considers hemodynamic stability, the severity of the injury and the presence of associated lesions. Non-operative management (NOM) is reserved for hemodynamically stable patients with minor duodenal injuries without perforation (AAST I/WSES I), as well as all duodenal hematomas (WSES I–II/AAST I–II) in the absence of associated abdominal organ injuries requiring surgical intervention. All hemodynamically unstable patients, those with peritonitis, or with CT findings consistent with duodenal perforations or AAST grade III or higher injuries are candidates for emergency surgery. If intervention is required, primary repair should be the preferred option whenever feasible, while damage control surgery is the best choice in cases of hemodynamic instability, severe associated injuries, or complex duodenal lesions. Definitive reconstructive surgery should be postponed until the patient has been adequately resuscitated. The role of endoscopic techniques in the treatment of duodenal injuries and their complications is expanding. **Conclusions**: Duodenal trauma is burdened by potentially high mortality. Among the possible complications, duodenal fistula is the most common, followed by duodenal obstruction, bile duct fistula, abscess, and pancreatitis. The overall mortality rate for duodenal trauma persists to be significant with an average rate of 17%. Future prospective research needed to reduce the risk of complications following duodenal trauma.

## 1. Background and Methods

The duodenum is an organ that is almost entirely retroperitoneal and relatively well protected, making traumatic injuries to it rare. Recent epidemiological studies reported that pancreatic-duodenal injuries account for 0.32% of all trauma cases and 2–4.7% of abdominal blunt and penetrating trauma; among these, 34.3% were identified as isolated duodenal injuries [1,2]. In the literature review by Santos et al. [3], the second portion accounted for 36% of duodenal injuries, followed by the third portion (18%) and the fourth portion (15%). First-portion duodenal injuries were the least prevalent (13%), while multiple portion injuries were observed in 18% of cases.

When duodenal trauma occurs, it can result in serious complications. This is primarily due to the duodenum’s proximity to major vascular structures such as the superior mesenteric artery and vein, which increases the risk of concomitant vascular injuries and hemorrhagic shock, as well as its intimate anatomical relationship with the pancreas, which increases the risk of pancreatic trauma and subsequent leakage of pancreatic enzymes, leading to retroperitoneal infection and necrosis. Moreover, duodenal repairs are associated with a higher failure rate compared to other segments of the intestine [4,5]. The diagnosis of duodenal trauma remains challenging. While penetrating injuries are most frequently identified during exploratory laparotomy, blunt trauma often lacks specific clinical manifestations. Physical examination may not be sufficient to establish clinical suspicion due to the retroperitoneal location of the duodenum [2]. Contrast-enhanced abdominal computed tomography (CT) currently represents the diagnostic modality of choice, even if imaging findings are frequently undetectable. Misclassification of injuries, delayed diagnosis, or late treatment can significantly raise morbidity and mortality [6,7].

The treatment of high-grade duodenal injury can be challenging because of its rarity and complex nature. A multidisciplinary approach is often necessary due to its correlation with concurrent injury to the pancreas, biliary tree, and major arterial systems. In the initial stages, this type of trauma is managed by intensive care physicians and emergency surgeons. When a conservative approach is pursued, endoscopists, interventional radiologists, and gastroenterologists are needed to improve outcomes and manage complications. In the later stages, the involvement of hepatobiliary surgeons could be essential for reconstructive surgery [8].

The present review integrates recent evidence (2015–2025), with particular emphasis on evolving diagnostic and therapeutic strategies, while preserving landmark studies that continue to inform contemporary clinical practice.

### Methods

This article is a narrative review of the current evidence on duodenal trauma. A literature search was performed in PubMed/MEDLINE and Scopus using combinations of the following keywords: “duodenal trauma”, “duodenal injury”, “pancreaticoduodenal trauma”, “non-operative management”, “damage control surgery”, “endoscopic management”, and “pediatric duodenal trauma”. We focused on articles published in English from 1980 to July 2025, although earlier landmark studies were included when they provided essential historical data or definitions. We included clinical studies, guidelines, systematic reviews, case series and relevant case reports addressing epidemiology, mechanisms of injury, diagnosis, management strategies and outcomes in adult and pediatric patients. Experimental studies, non-traumatic duodenal disease and non-English publications were excluded. Because of the heterogeneity and rarity of duodenal trauma, no formal systematic review or meta-analysis was attempted.

The literature search yielded 658 records across PubMed/MEDLINE and Scopus. After removal of duplicates (*n* = 148), 510 articles underwent title and abstract screening. Of these, 438 were excluded based on the predefined criteria, and 72 studies were ultimately included in this narrative review.

## 2. Mechanism of Injury

Duodenal injuries can result from blunt or penetrating trauma. Blunt trauma occurs due to a direct blow to the epigastric region from a narrow object or due to a sudden increase in intraluminal pressure [2]. Isolated duodenal injuries are relatively uncommon because the duodenum is situated next to biliary and circulatory structures, as well as the pancreas. When associated with pancreatic injuries, they are typically caused by severe anteroposterior compression trauma against the spine, mostly related to seat belt injuries or deceleration injuries [6]. Blunt compression or deceleration, commonly seen after motor vehicle collisions or direct epigastric impact, often leads to intramural hematoma, contusion, or transection at relatively fixed segments of the duodenum (D1–D3), while penetrating mechanisms including stab or gunshot wounds more frequently result in focal perforation and free retroperitoneal or intraperitoneal air [9,10]. Traumatic duodenal injuries are rare in children, occurring in less than 1% of all pediatric trauma cases and in 2–10% of children presenting with abdominal trauma [11,12]. The most common mechanisms are motor vehicle collisions, falls from height, or blows against bicycle handlebars [13,14,15], and less frequently assaults, child abuse and non-accidental trauma, such as endoscopic biopsy procedures and foreign body ingestions [15,16,17,18,19,20]. Pediatric anatomy predisposes children to duodenal injuries from blunt trauma due to reduced intra-abdominal fat and limited protection from a more horizontally oriented costal margin [21]. Penetrating modalities account for the majority of injuries in the adult population, accounting for 53.6–90% of cases [22,23,24], with gunshot wounds being responsible for about 70–80% of these occurrences, especially in countries with high rates of civil violence. This type of injury is associated with poorer clinical outcomes compared to blunt trauma [2,25]. The outcome of duodenal trauma shows significant divergence between adult and pediatric populations, due to the underlying etiology and the prevailing mechanisms of injury. In pediatric patients, the injury is almost exclusively due to blunt trauma, resulting in a high incidence of intramural duodenal hematoma associated with very low overall mortality rate and high healing rates with non-operative management, showing efficacy in up to 94% of cases [26]. Instead, in adults, duodenal trauma is more frequently caused by penetrating trauma or high-energy blunt mechanisms such as motor vehicle collisions, leading to a greater prevalence of high-grade duodenal lesions and associated vascular or pancreatic injuries that necessitate complex surgical intervention and result in a significantly higher mortality rate, between 15 and 30% when severe injuries are detected [8,27,28]. A higher prevalence of duodenal injuries is observed in males within both adult and pediatric patient groups [13]. Duodenal trauma has a high mortality rate and, in the literature, it varies greatly, ranging from 9.7% to 21% [29,30,31]. In a recent multicenter study, about 70% of patients had associated intra-abdominal damage. The cause of immediate death is typically hemorrhagic shock from these related injuries rather than the duodenal damage itself [32]. Late mortality is predominantly linked with septic complications, most commonly resulting from the development of fistulas and abscesses [25]. In a study about penetrating duodenal trauma patients in the National Trauma Data Bank, Phillips et al. [33] found that the likelihood of death following penetrating duodenal injuries was predicted by lower initial systolic blood pressure, lower Glasgow Coma Scale (GCS) score, higher pulse, higher Injury Severity Score (ISS), and higher OIS grades.

## 3. Classification

The 2019 World Society of Emergency Surgery (WSES)-American Association for the Surgery of Trauma (AAST) guidelines [8] recommend incorporating both the AAST-OIS grading (Table 1) and the patient’s hemodynamic status to obtain an adequate duodenal injuries classification system (Table 2), which stratifies duodenal injuries into four categories:

▪ Minor injuries:
  ▪ WSES class I: includes hemodynamically stable patients with AAST-OIS grade I duodenal lesions.
▪ Moderate injuries:
  ▪ WSES class II: includes hemodynamically stable patients with AAST-OIS grade II duodenal lesions.
▪ Severe injuries:
  ▪ WSES class III: includes hemodynamically stable patients with AAST-OIS grade III–IV–V duodenal lesions.
  ▪ WSES class IV: includes hemodynamically unstable patients with AAST-OIS grade I–V duodenal lesions.

Since duodenal trauma is most often associated with other injuries, especially to the pancreas or the biliary tract, the overall injury grade is determined by the most severe injury among the duodenal, pancreatic, and extrahepatic biliary components [8].

## 4. Diagnosis

The diagnostic approach to suspected duodenal trauma involves a combination of clinical assessment, laboratory investigations, radiological imaging and, in selected cases, surgery. Prompt diagnosis is critical but frequently challenging due to the retroperitoneal location of the duodenum and the possible absence of clear initial clinical signs. The interval between the traumatic event and definitive management plays a critical role in patient outcomes; delays exceeding 24 h are strongly associated with a higher incidence of postoperative complications and a significant rise in mortality, with an overall mortality ranging from 5 to 30% [34].

### 4.1. Clinical Presentation

When a patient with abdominal trauma, either isolated or associated with other body injuries, is admitted to the emergency room, the first step is the immediate assessment for life-threatening injuries and this is achieved during the primary survey using the ABCDE approach established by the Subcommittee on Advanced Trauma Life Support (ATLS) of the American College of Surgeons (ACS), Committee on Trauma [32]. Hemodynamic stability must be assessed immediately, as signs of shock, ongoing hemorrhage, or peritonitis may necessitate urgent surgical intervention without delay for advanced investigations. A careful evaluation of the trauma mechanism is crucial, since it provides important insight into both the probability and the expected pattern of injury. At this time, it is possible to carry out the clinical evaluation of the abdomen and perform laboratory tests, extended focused assessment with sonography for trauma (E-FAST), and chest and pelvic X-ray. Symptoms of duodenal trauma are often nonspecific, especially in cases of blunt trauma, where there may be a latency period between the traumatic event and symptom onset. In penetrating trauma, suspicion arises primarily from the injury mechanism and trajectory of the penetrating object [4,8,35]. Common clinical findings include abdominal pain, especially in the epigastric or periumbilical region, nausea and vomiting, possibly bilious or bloody, and signs of peritoneal irritation which frequently indicate intraperitoneal perforation. Ecchymosis over the flanks or back is a clinically relevant marker of blunt abdominal trauma.

### 4.2. Laboratory Findings

Although nonspecific, laboratory tests may provide indirect evidence and can provide supportive information. Blood tests that are usually performed initially are blood gas analysis (ABG) to evaluate acid-base balance, Lactate and Base Excess (BE), complete blood count (CBC), electrolytes, renal function, blood typing, partial Thromboplastin Time (PTT) and activated Partial Thromboplastin Time (aPTT). A complete blood count can offer useful insights in patients with duodenal trauma. An elevated white blood cell count may reflect an underlying inflammatory or infectious process, whereas reduced hemoglobin levels could point to active bleeding. Nonetheless, these laboratory abnormalities are not specific for duodenal injury and must always be interpreted alongside the patient’s clinical condition and imaging findings. Elevated serum amylase or lipase may be suggestive of associated pancreatic injury, although their role in diagnosing duodenal trauma is limited. Liver function tests and coagulation profiles are important in patients with concomitant liver injury.

### 4.3. Radiological Imaging

The Extended focused assessment with sonography for trauma (E-FAST) is usually the initial imaging test, given its speed, non-invasive nature, and ability to be repeated easily at the bedside, making it ideal in emergency situations. Although it is not used to evaluate retroperitoneal structures, it is highly sensitive for identifying intraperitoneal free fluid. Chest X-ray can be useful in identifying the presence of a subdiaphragmatic air caused by bowel or gastric perforation. For this reason, in hemodynamically stable patients, contrast-enhanced multidetector abdomen computed tomography (MDCT) is considered the gold standard for diagnosis. In patients who are hemodynamically unstable and suspected of having intra-abdominal injuries, an emergency laparotomy is required without delay for further imaging. Esophagogastroduodenoscopy (EGDS) can detect mucosal injuries in stable patients but is contraindicated in suspected perforation [36,37,38].

MDCT is the most frequently used imaging modality to assess abdominal trauma and has a sensitivity of 64% and specificity of 97% for bowel injury [39,40]. The use of intravenous contrast improves visualization of key findings such as wall disruption, periduodenal hematoma, retroperitoneal air, and injuries to adjacent structures including the pancreas and mesentery. Among radiological features that may indicate duodenal injury are duodenal wall thickening (>4 mm), fat stranding, hyperattenuating intramural hematoma, sometimes with associated luminal narrowing, the presence of fluid accumulation adjacent to the duodenum or within the right anterior pararenal space, and reduced contrast enhancement of the bowel wall. Pancreatic contusions are frequently associated with abdominal blunt trauma and must be carefully sought [9,37,38].

Importantly, reported diagnostic accuracy varies according to injury severity. In high-grade injuries with direct radiological signs such as mural discontinuity or contrast extravasation, MDCT demonstrates very high sensitivity and specificity (around 95%) for detecting perforation and excluding injuries requiring surgery. Conversely, in low-grade blunt injuries, the sensitivity may be lower because only subtle or indirect findings are present, which explains why an initially negative CT does not rule out evolving duodenal injury [36,41,42].

The differential diagnosis in this setting is important: while an intramural hematoma typically appears as a homogeneous high-attenuation lesion without mural discontinuity, a duodenal contusion can show less well-defined thickening. The high sensitivity (95%) and the high specificity of abdominal MDCT (95–99.6%) in excluding signs of perforation (free air, contrast extravasation) translate into an extremely high negative predictive value (NPV) for ruling out injuries requiring surgical management. Therefore, abdominal CT confirms high reliability in identifying hematoma-based, minor or moderate-grade injuries (WSES I–II / AAST I–II) and differentiating them from higher-grade WSES/AAST (III-V) [8,36,41]. In cases of pancreatic injury, secondary duodenal wall edema may mimic primary duodenal trauma, and only careful assessment of the pancreas allows the radiologist to clarify the origin. The most indicative features of duodenal perforations are discontinuity of the intestinal wall with the presence of extraluminal air or contrast leakage [4,8,42]. High-speed collisions, particularly those involving seat belts, may generate shearing forces at the fixed portions of the duodenum, most commonly the second and third segments. CT may demonstrate wall disruption, retroperitoneal hematoma, fluid collections, or active extravasation of contrast. Spinal fractures, especially at the thoracolumbar junction, are frequently associated and should heighten suspicion. In this context, the radiologist must decide whether a lesion represents a simple hematoma or a full-thickness transection. Multiplanar reconstructions are often required to identify wall defects and to differentiate duodenal injury from concomitant mesenteric or jejunal lesions [9]. In penetrating trauma such as stab and gunshot wounds, CT can demonstrate a focal mural defect, extraluminal gas, localized retroperitoneal or intraperitoneal fluid, and in some cases the trajectory of the foreign body itself. Although CT may underestimate small perforations, it remains an essential tool for triage and for the evaluation of associated abdominal injuries. The main diagnostic challenge lies in identifying duodenal perforation and injury to adjacent small bowel segments. Oral contrast leakage or careful evaluation of the wound trajectory can help localize the site of perforation. In contrast, iatrogenic perforations may be indistinguishable radiologically, and a precise clinical history is critical for interpretation [42]. Iatrogenic duodenal injuries may also occur after endoscopic procedures such as ERCP or biopsy, or as postoperative complications. Any defect identified during endoscopy that is suitable for endoscopic closure must be sealed immediately. On CT, these lesions present with mural thickening, retroperitoneal or intramural air, or minor oral contrast leaks. These findings can mimic those of blunt or penetrating trauma, but correlation with recent medical or surgical interventions usually clarifies the diagnosis. It is also important to recognize that limited retroperitoneal air in the immediate postoperative period may be expected and should not be mistaken for pathological leakage [10].

When CT results are doubtful or equivocal, further diagnostic strategies should be tailored according to the patient’s hemodynamic status and clinical evolution. In hemodynamically stable patients who continue to raise clinical concern despite an initial CT showing negative or nonspecific findings, repeating CT after 12–24 h may reveal evolving features of injury that were initially occult. In these delayed cases, significant local contamination and septic signs may already be present due to the evolving injury. Alternatively, an upper gastrointestinal contrast study with water-soluble contrast can be employed to assess for subtle leaks. Magnetic resonance imaging (MRI) or magnetic resonance cholangiopancreatography (MRCP) may serve as useful adjuncts, particularly as secondary, non-invasive tools for evaluating pancreatic or biliary involvement, and are especially valuable in pediatric or pregnant patients where radiation exposure should be minimized [43,44,45].

## 5. Management

The management of duodenal trauma requires a multimodal approach that considers hemodynamic stability, the severity of the injury and the presence of associated lesions, involving the expertise of multiple specialists. In general, treatment can be non-operative in selected stable patients with minor injuries or it can be surgical in more severe or complicated cases. In recent years, there has been a trend toward less invasive strategies, with the aim of reducing complications and improving outcomes.

### 5.1. Antibiotic Therapy

Currently, there are no studies specifically addressing antibiotic therapy for duodenal trauma. The 2023 WSES guidelines on the management of trauma in elderly and frail patients [46] recommend antibiotic prophylaxis covering both aerobic and anaerobic bacteria in all elderly and frail patients with penetrating abdominal trauma. They also advise empiric antibiotic therapy for elderly patients presenting with sepsis or septic shock, or with additional risk factors such as obesity, immunosuppression, or a high ASA score. The recommended agents should target the most common pathogens responsible for intra-abdominal infections, including Escherichia coli, other Enterobacterales, and Clostridiales. Currently, there is no evidence supporting the use of antibiotics beyond 24 h outside these specific clinical scenarios. A 2019 Cochrane review [47] highlighted the lack of randomized controlled trials, demonstrating the effectiveness of antibiotic prophylaxis in penetrating abdominal trauma, with available studies being of very low quality. In the same year, Herrod et al. [48] reviewed the literature to assess the optimal choice and duration of antibiotic therapy. Although the evidence was weak and at high risk of bias, the review suggested that the duration of prophylaxis does not appear to significantly influence the reduction in infectious complications. However, no definitive conclusions could be drawn regarding the most appropriate antibiotic regimen.

### 5.2. Non-Operative Management

Non-operative management (NOM) is reserved for hemodynamically stable patients with minor duodenal injuries, in the absence of associated abdominal organ injuries requiring surgical intervention [8]. Minor duodenal injuries include non-transmural duodenal lesions without perforation (AAST I/WSES I), as well as all duodenal hematomas (WSES I–II/AAST I–II). Conservative treatment consists of bowel rest and nasogastric decompression and serial abdominal examinations. Current literature supports the early initiation of enteral nutrition as soon as a decrease in nasogastric output is observed. If enteral feeding cannot be initiated within 7 days, parenteral nutrition is recommended [49]. Some authors recommend performing an oral water-soluble contrast study—typically after 5 to 7 days—prior to nasogastric tube removal and resumption of oral intake, to exclude persistent obstruction or the presence of a delayed fistula. If duodenal obstruction does not resolve within two weeks, conservative management should be considered unsuccessful. In such cases, surgical intervention is recommended to rule out concomitant lesions of the pancreatic head that may contribute to the persistence of the stenosis [5,50,51] A 2016 study by Bradley et al. [52] analyzed the role of conservative management in patients with indirect signs of duodenal injury on abdominal CT. Their findings demonstrated that isolated periduodenal hematoma or fluid collections are not, in themselves, clear indications for laparotomy and the patients without clinical signs suggestive of progression can be observed with serial clinical and radiological assessments. Although the failure rate of conservative management in these cases was approximately 20%, only 5% of patients ultimately required exploratory laparotomy for a duodenal injury.

### 5.3. Operative Management

**Figure 1:**
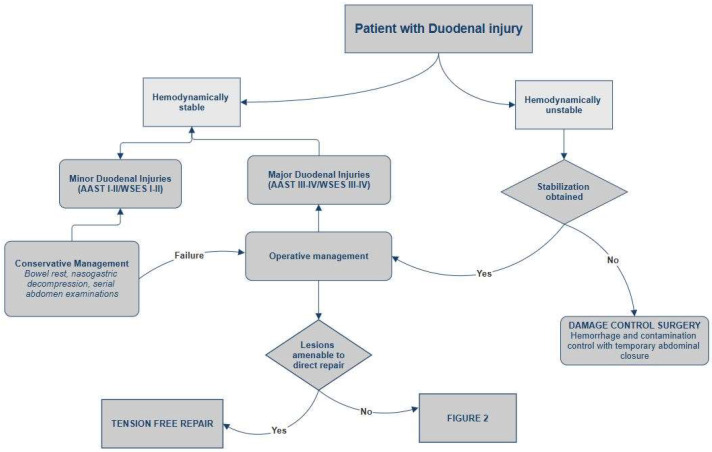
Management of duodenal trauma.

**Figure 2:**
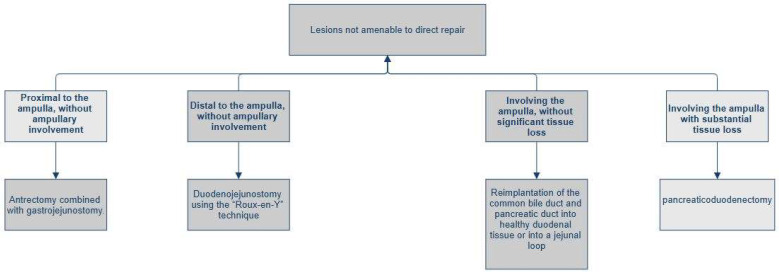
Management of duodenal trauma not amenable to direct repair.

All hemodynamically unstable patients, those with peritonitis, or with CT findings consistent with duodenal perforations or AAST grade III or higher injuries are candidates for emergency surgical intervention [8] (Figure 1 and Figure 2). In cases of hemodynamic instability with physiological impairment, Damage Control Surgery (DCS) should be performed, focusing on control of hemorrhage and contamination through rapid injury repair, without restoring intestinal continuity, and with temporary abdominal closure. Patients are subsequently transferred to the intensive care unit for optimization of physiological parameters and, after stabilization, undergo definitive surgical intervention [52,53,54]. A systematic review analyzed data from 39 studies on the indications for DCS, identifying only few conditions in which the use of this technique improved patient survival: hypothermia, acidosis, development of coagulopathy during surgery, and the presence of two concomitant injuries that could not be definitively repaired in a timely manner [55]. In other patients requiring laparotomy, in the absence of the previously mentioned conditions, the surgery should aim for rapid primary repair of the injury. In case of incidental findings of minor injuries such as serosal lesions or duodenal hematomas during trauma laparotomy, surgical exploration is indicated. To ensure optimal anatomical exposure, it is recommended to perform the Kocher maneuver for mobilization and inspection of the second portion of the duodenum, the Cattell maneuver for access to the third portion, and division of the ligament of Treitz for complete visualization of the fourth portion, allowing thorough assessment of the duodenum up to its transition with the jejunum [56]. Once complete exposure is achieved, full-thickness duodenal perforation must be excluded, as it requires primary repair. In the case of a non-expanded duodenal hematoma occupying more than 50% of the duodenal lumen, evacuation via incision of the duodenal wall with meticulous hemostasis is recommended. If hematoma involves less than 50% of the lumen, a conservative approach may be considered. In all cases, placement of a jejunostomy or a jejunal feeding tube for enteral nutrition is indicated [4,5]. If, during exploration, duodenal injuries of AAST grade III or higher are found, studies over the past thirty years on duodenal trauma have demonstrated that, when feasible, simple tension-free repair combined with external drainage and decompression via a nasogastric tube represent the most effective treatment. The primary repair involves meticulous debridement to ensure clean, viable edges, followed by transverse closure of the perforation (to prevent stenosis), performed in either a single or double layer according to the surgeon’s preference [4,31,53,54,56,57,58]. Ancillary techniques—such as pyloric exclusion, duodenal diverticulization, and duodenostomy tube placement—traditionally employed to reduce enzymatic fluid flow over primary repair sites, have largely fallen out of favor. In 2023, García et al. [58] published a multicenter retrospective review analyzing data from 288 patients with duodenal trauma. The study demonstrated that patients who underwent primary repair had a lower incidence of duodenal leaks (DLs), identifying complex surgical procedures as an independent risk factor for DL. In the same year, Choron et al. published a multicenter study on patients with duodenal trauma complicated by postoperative duodenal leak, showing that ancillary procedures do not prevent DL formation and do not have a positive impact on morbidity and mortality in patients with leaks [8,59]. However, due to the fixed anatomical position of the organ, tension-free repair may not be achievable in certain cases, even when the injury involves less than 50% of the organ’s circumference. Such cases require more complex procedures tailored according to the location of the duodenal injury. In patients with lesions not amenable to direct repair or resection with duodeno-duodenal anastomosis, the surgical options include [4,5,34,53,54]:

▪ Lesion proximal to the ampulla, without ampullary involvement: antrectomy combined with gastrojejunostomy.
▪ Lesion distal to the ampulla, without ampullary involvement: duodenojejunostomy using the “Roux-en-Y” technique.
▪ Lesion involving the ampulla or distal common bile duct (AAST Grade IV), without significant tissue loss: complex reconstruction, which may include the reimplantation of the common bile duct (via choledochoduodenostomy or choledochojejunostomy on a Roux-en-Y limb) and peripancreatic drainage [32].
▪ Lesion involving the ampulla with substantial tissue loss: indication for pancreaticoduodenectomy (PD).

With regard to pancreatoduodenectomy (PD) in the setting of trauma, the current literature recommends, in most cases, a two-stage approach, with an initial procedure aimed at debridement, source control, and, if necessary, the placement of devices for enteral nutrition. The availability of surgeons with specific expertise in hepatobiliary surgery appears to be associated with better outcomes and should be considered according to the complexity of the case [8,53]. However, a meta-analysis published in 2020, including 22 studies on the outcomes of traumatic pancreatoduodenectomy in patients with AAST grade IV and V injuries, found that performing the procedure in two stages does not confer an advantage in terms of mortality compared with a single-stage approach in hemodynamically stable patients (mortality rate 38.7% vs. 34.2%) [60]. On the contrary, in stable patients, a statistically significant difference was observed in favor of single-stage PD, with a survival rate of 85.4%. These findings are corroborated by a recent retrospective study (2024) conducted on a cohort of 20 consecutive patients with major pancreatoduodenal trauma. According to this analysis, the primary cause of death within the first 24 h in patients undergoing PD was acute hemorrhage due to associated vascular injuries. In individuals who maintained hemodynamic stability during the initial operation, single-stage pancreatoduodenectomy proved to be safe, avoiding the surgical and physiological stress of a second operative stage [61].

### 5.4. The Expanding Role of Endoscopic Management

Recent studies highlight the expanding role of endoscopy in the multidisciplinary management of duodenal trauma, transitioning from a diagnostic and supportive tool to a definitive therapeutic one [62]. Although the current literature primarily consists of case reports and case series regarding cases of duodenal lesions of different etiologies, growing scientific interest and novel technological developments suggest a progressive evolution of endoscopic techniques and an increasing range of potential applications in this complex field. Evidence suggests that endoscopy is particularly beneficial in managing less severe lesions, according to the WSES/AAST classification, and their associated complications. Duodenal intramural hematomas (DIHs) (WSES I-II/AAST I-II) resulting from blunt abdominal trauma are primarily managed non-operatively. However, these lesions can lead to persistent obstruction, in which case endoscopy offers alternative solutions that prevent the need for surgical intervention. Some case reports suggest the use of endoscopic balloon dilation to resolve the obstruction caused by duodenal hematoma [63]. For example, in the case report by Alharbi, Fahad M et al. [64], the authors described a pediatric patient with a 27-day persistence of intestinal obstruction symptoms after a road traffic accident, caused by a DIH in the third portion of the duodenum causing narrowing of the duodenal lumen. Although this endoscopic procedure is not widely used and not standardized, DIH was successfully managed via esophagogastroduodenoscopy (EGD) with endoscopic balloon dilatation, avoiding the need for surgical intervention. Conversely, Valerii et al. [65] reported a case of intramural spontaneous hematoma with symptoms of intestinal obstruction treated with the application of Endoscopic Ultrasound (EUS)-guided drainage techniques through the placement of Lumen-Apposing Metal Stents (LAMSs), to support continuous drainage of the hematoma into the lumen. For more complex, high-grade lesions (WSES III-IV/AAST III-IV), surgical repair remains the gold standard of treatment. Nevertheless, endoscopy can significantly benefit the management of duodenal injury in both the intraoperative and post-operative settings. For instance, during surgery to repair a complex duodenal injury, intraoperative endoscopy is highly valuable both for more accurate lesion localization, preventing delayed detection of missed injuries, and as a supportive tool for the optimal placement of a naso-jejunal feeding tube [66]. This tube is critical for ensuring post-operative enteral nutrition and providing mechanical protection to the duodenal repair site. Post-operatively, it is not uncommon to face the complex issue of biliary fistula formation, where endoscopy may represent a valuable alternative to surgical re-intervention in selected cases. For post-operative fistulas resulting from small leaks at the suture line or minor residual defects, therapeutic endoscopy employing Over-The-Scope Clips (OTSCs) or direct endoscopic suturing can be a viable option [67,68]. Conversely, for complex injuries associated with high-output biliary fistulas, a more advanced procedure such as Endoscopic Ultrasound-guided Choledochoduodenostomy (EUS-CDS) with endoscopic nasobiliary drainage (ENBD) may be considered to provide effective biliary diversion, avoiding the morbidity associated with complex reconstruction [69].

## 6. Complications

Complications related to duodenal trauma can be broadly classified into those directly caused by the injury and those secondary to operative or non-operative management. The former mainly include intra-abdominal infections and mechanical obstruction due to mural edema, intramural hematoma, or post-traumatic stricture. Delayed diagnosis continues to be associated with higher rates of intra-abdominal and septic complications [70].

Among treatment-related complications, duodenal fistula or leak is the most clinically relevant and represents a major source of postoperative morbidity. Although historically complex surgical strategies (e.g., diverticulization, pyloric exclusion, tube duodenostomy) were advocated to prevent leaks, growing evidence shows that they do not reduce fistula rates and may increase morbidity and mortality [54,58]

A large multicenter Panamerican Trauma Society study confirmed this trend, reporting that primary repair was the most common operative strategy (80%) and was associated with a significantly lower leak rate across all AAST injury grades, supporting primary repair as the treatment of choice even in complex injuries [32].

Recent multicenter studies confirm that procedures other than primary repair are independently associated with an increased risk of leak [58].

Current evidence suggests that complex procedures do not improve outcomes, whereas primary repair is associated with fewer gastrointestinal complications [50,59]. Management of duodenal fistula typically focuses on drainage and sepsis control, and retroperitoneal laparostomy has been reported as an effective alternative to traditional anterior drainage, with a substantial proportion of leaks closing spontaneously [54].

## 7. Conclusions

Duodenal injury is a challenging concern in patients with blunt or penetrating trauma. Although uncommon, it is burdened by potentially high mortality due to difficult early diagnosis and the rate of delayed treatment, common associated injuries and possible complications. The overall mortality rate for duodenal trauma persists to be significant with an average rate of 17% [71]. The choice between non-operative management and surgical treatment depends on the overall clinical scenario in addition to the size and the timing of recognition. If surgical management is needed, primary repair should be the preferred option whenever possible, while damage control surgery is the best choice in cases of hemodynamic instability, serious associated injuries or complex duodenal injury. Definitive reconstructive surgery should be postponed until the patient has been adequately resuscitated, respecting, when possible, the general rule of “Less is Better” [30]. If an iatrogenic injury is spotted during an endoscopic procedure, prompt closure may eliminate the need for surgical intervention, whereas delays in recognition can lead to worse outcomes for the patient; improving novel endoluminal techniques and endoscopic devices could progressively increase the endoscopic role in the future management of intestinal perforations [69]. Thus, the most serious complication following the surgery is duodenal fistula, with an average incidence for 6.6% as reported by Asensio et al. [72], but other possible complications include duodenal obstruction, bile duct fistula, abscess and pancreatitis. Improved outcomes for duodenal trauma are correlated with prompt recognition and timely intervention. Future prospective research could be helpful to further optimize patient care and significantly reduce the risk of complications following duodenal trauma.

## Figures and Tables

**Table 1:** AAST duodenum injury scale. D1: first portion of duodenum; D2: second portion of duodenum; D3: third portion of duodenum; D4: fourth portion of duodenum.

AAST Duodenum Injury Scale		
Grade	Type of Injury	Description
I	Hematoma	Involving single portion of duodenum
	Laceration	Partial thickness, no perforation
II	Hematoma	Involving more than one portion
	Laceration	Disruption <50% of circumference
III	Laceration	Disruption 50–75% of circumference of D2 Disruption 50–100% of circumference of D1, D3, D4
IV	Laceration	Disruption >75% of circumference of D2 Involving ampulla or distal common bile duct
IV	Laceration	Massive disruption of duodenopancreatic complex
	Vascular	Devascularization of duodenum

**Table 2:** WSES classification of duodenal injury.

WSES Classification Of Duodenal Injuries					
Grade	WSES Class	AAST	Hemodynamic Status	Type Of Injury	Description
Minor	WSES Class I	I	Stable	Hematoma	Involving single portion of duodenum
				Laceration	Partial thickness, no perforation
Moderate	WSES Class II	II	Stable	Hematoma	Dnvolving more than one portion
				Laceration	Disruption <50% of circumference
Severe	WSES Class III	III	Stable	Laceration	Disruption 50–75% of circumference of d2 Disruption 50–100% of circumference of d1, d3, d4
		IV	Stable	Laceration	Disruption >75% of circumference of d2 involving ampulla or distal common bile duct
		V	Stable	Laceration	Massive disruption of duodenopancreatic complex
				Vascular	Devacularization of duodenum
	WSES Class IV		Unstable	Any	Any

## Data Availability

No new data were created or analyzed in this study.
